# Effect of calcium and magnesium on starch synthesis in maize kernels and its physiological driving mechanism

**DOI:** 10.3389/fpls.2023.1332517

**Published:** 2024-01-08

**Authors:** Zhaoquan He, Xue Shang, Tonghui Zhang, Jianying Yun

**Affiliations:** ^1^ School of Life Sciences, Yan’an University, Yan’an, China; ^2^ Shaanxi Key Laboratory of Chinese Jujube, Yan’an University, Yan’an, China; ^3^ College of Land Resource and Environment, Jiangxi Agricultural University, Jiangxi, Nanchang, China; ^4^ Northwest Institute of Eco-Environment and Resources, Chinese Academy of Sciences, Lanzhou, China

**Keywords:** calcium and magnesium, kernel starch, endogenous hormone, enzymes of STC synthesis, physiological mechanism

## Abstract

The content of kernel starch (STC), which is a fundamental indicator of the nutritional value of maize, is directly correlated with the grain’s taste and aroma. Both calcium (Ca) and magnesium (Mg) are critical nutrients that play a significant role in the growth and development of maize, as well as in the synthesis of STC. To determine the physiological driving mechanisms of Ca and Mg effects on the accumulation of STC synthesis in maize kernels and the characteristics of their effects on endogenous hormones and enzymes of STC synthesis in maize leaves, our study applied foliar Ca and Mg fertilizers at various levels to maize prior to pollination. (1) The levels of Ca, Mg, indole-3-acetic acid (IAA), gibberellin (GA), and zeatin riboside (ZR) in maize leaves increased and then decreased after the supplementation of Ca and Mg. They peaked on the 32nd day after pollination. In contrast, the levels of abscisic acid (ABA) initially decreased and then increased. Ca and Mg had a negative correlation with ABA and a positive correlation with IAA, GA, and ZR. (2) As the levels of Ca and Mg increased, correspondingly rose the activities of enzymes responsible for STC synthesis and the content of STC and its components. Principally influencing the synthesis of STC were ABA, IAA, uridine diphosphate-glucose pyrophosphorylase (UDPG), granule-bound starch synthase (GBSS), and soluble starch synthase (SSS). (3) “IAA–UDPG or GBSS–STC” was the predominant physiological regulation pathway of Ca on kernel STC, whereas “IAA–GBSS–STC” was the dominant physiological regulation pathway of Mg on kernel STC. The regulatory impact of STC by UDPG and GBSS was positive, as were the effects of IAA on UDPG and GBSS. In conclusion, the accumulation of kernel starch was significantly enhanced by Ca and Mg supplementation via the modulation of endogenous hormone levels and key enzyme activities. This research identifies a viable approach to improve the nutritional composition of maize.

## 1 Introduction

Constantly utilized as a food, feed, and industrial raw material, maize is one of the most essential food crops in the world. In maize kernels, STC is a significant nutrient source (Mancebo et al., 2015). Glucose is supplied to germinating seedlings and developing embryos to maintain their normal metabolic activities and to regulate the kernel’s size, texture, and nutritional quality (Wasserman et al., 2021). Furthermore, STC constitutes a significant carbohydrate source in both animal and human food. Ca and Mg are crucial nutrients for the development and growth of crops. Ca and Mg can control kernel STC synthesis in maize via physiological mechanisms, including the regulation of membrane permeability, activation of hormonal components and enzyme systems, and maintenance of cellular osmotic pressure (Abadi et al., 2020; Zhang G. S. et al., 2020). Hence, an investigation into the regulation of STC synthesis and accumulation in maize kernels by Ca and Mg can contribute to a more comprehensive comprehension of the maize growth and development mechanism, as well as furnish a theoretical foundation and technical assistance for the enhancement of maize quality (Buchelt et al., 2020).

Endogenous hormones play a crucial role as signaling molecules within crops, governing a multitude of growth and development processes. Previous research has demonstrated that endogenous hormones can regulate the expression of genes associated with STC synthesis in maize kernels, thereby influencing the synthesis and accumulation of STC (Ahmad et al., 2019; Zhang T., et al., 2020). Such as, GA can increase the rate of STC synthesis by stimulating the expression of genes involved in starch synthesis. Furthermore, the stability and activity of enzymes involved in STC synthesis can be influenced by hormones, which in turn impacts the synthesis and accumulation of STC (Ahammed et al., 2020). STC synthesis in maize kernels involves Ca and Mg in conjunction with endogenous hormones in an interactive manner. Liu et al. (2018) discovered that the synthesis and signaling of endogenous hormones can be influenced by Ca and Mg, which regulate the activity of STC synthase and the expression of genes associated with STC synthesis. Additionally, endogenous hormones can influence the accumulation and distribution of Ca and Mg in maize kernels by regulating their uptake and transport. This finding provides compelling evidence that the interaction between endogenous hormones and Ca and Mg is a significant regulator of STC synthesis in maize kernels (Lando et al., 2020). Hence, conducting a comprehensive investigation into the interplay and mechanisms involving endogenous hormones, Ca, and Mg in the synthesis of STC from maize kernels holds substantial theoretical and practical importance. Such research would unveil the regulatory network governing STC synthesis and contribute to the enhancement of maize quality (Li et al., 2018).

The enzymes responsible for STC synthesis comprise a consortium that functions synergistically throughout the process, cooperating to achieve the formation and retention of STC molecules (Li et al., 2014). Enzymes involved in STC synthesis typically collaborate in a particular sequence to progressively produce STC molecules (Ding et al., 2009). At the outset, amylose is formed when adenosine diphosphate-glucose pyrophosphorylase (ADGP) catalyzes the reaction between glucose-1-phosphate and adenosine diphosphate (ADP) glucose (Li et al., 2021). Starch synthase (SS) then increases the length of existing STC granules by adding glucose molecules. The formation of branches in the STC molecule is the joint effort of GBSS and starch branching enzyme (SBE) (Baranov et al., 2014). Enzymes involved in STC synthesis collaborate synergistically to guarantee the correct formation of the STC molecule. An illustration of this is how the activity of ADGP influences the activities of SS and SBE (Ballicora et al., 2004). SS necessitates the provision of an appropriate quantity of ADP glucose, while SBE relies on the branch initiation sites produced by GBSS to add branches. Enzymes of this nature are crucial in the maize STC biosynthesis pathway, guaranteeing the synthesis and accumulation of STC so that carbon sources and energy can be released from maize when necessary (He et al., 2020). Numerous studies have demonstrated that Ca and Mg can regulate the synthesis and accumulation of STC in maize by influencing the activity and expression of enzymes involved in STC synthesis (Rehman et al., 2018). Ca and Mg, for instance, are regarded as cofactors of enzymes involved in STC synthesis and can bind to the active site of said enzymes to aid in the catalytic process. Enzymes involved in STC synthesis may experience an increase in catalytic activity due to conformational or charge state modifications caused by the binding of Ca and Mg (Lambers and Barrow, 2020). In addition, Ca and Mg are also implicated in the regulation of genes encoding enzymes that facilitate STC synthesis. They influence the binding capacity or activity of transcription factors, which in turn govern the levels of gene expression and promoter activity in enzymes responsible for STC synthesis. This has an impact on the enzyme accumulation during STC synthesis (Liu et al., 2018). Furthermore, metabolic pathways linked to the enzymes responsible for STC synthesis, including sugar synthesis and transport, can be influenced by Ca and Mg. Their potential involvement in enzyme-substrate translocation and metabolism could have an impact on the accessibility of substrates for the enzymes responsible for STC synthesis. This regulates the activity of enzymes involved in STC synthesis and the STC synthesis process as a whole (Tang and Luan, 2017). Nevertheless, the precise regulation of STC synthesis enzyme activity by Ca and Mg may be compromised. An imbalance in the concentrations of Ca and Mg can lead to perturbed enzyme activity during STC synthesis, thereby influencing the overall process (Kong et al., 2020). Meanwhile, phytohormones have a substantial impact on the activity and expression of enzymes involved in STC synthesis. Moreover, the regulation of these enzymes is altered through complex interactions between Ca and Mg supplementations and phytohormones (Rashid et al., 2020; Zhang et al., 2020). To achieve the objective of more precise and efficient regulation of maize STC accumulation, the quantification of Ca and Mg supplements and the mastery of their multiple interactions with phytohormones and enzymes of STC synthesis must be addressed further in the study.

Northwest China’s major rain-fed spring maize-producing region is the Loess Plateau. When considering the provision of Ca and Mg for maize absorption, the substitutional states exhibit the highest efficiency. Ca substitutional levels in the farmland soils of the Loess Plateau are 3,338 mg kg^-1^, while Mg substitutional levels are 282 mg kg^-1^ (Wang et al., 2020). Nevertheless, for continuous maize growth, it is critical to apply Ca and Mg supplements containing 5000 mg kg^-1^ and 2000 mg kg^-1^, respectively (Cakmak and White, 2020). This observation suggests that the efficient levels of Ca and Mg in this region have substantially reduced the minimum thresholds necessary for normal maize development and growth. Considering the inadequate levels of Ca and Mg fertility on farmland located on the Loess Plateau, the objective of our research was to examine how Ca and Mg supplementations affect the regulation of STC synthesis and accumulation in maize kernels in rain-fed arid regions of the Loess Plateau. The study primarily focused on the following inquiries: (1) to examine the interplay between Ca and Mg and endogenous hormones, as well as to quantify the impact of varying levels of Ca and Mg on the synthesis and accumulation of STC in maize kernels; (2) to elucidate the function of Ca and Mg in controlling the activity of enzymes responsible for STC synthesis and to identify the mechanism that governs the “Ca and Mg–endogenous hormones–enzymes of STC synthesis–STC level” of the kernels. This provides crucial theoretical support for elucidating the regulatory network underlying STC synthesis in maize kernels and enhancing maize quality.

## 2 Materials and methods

### 2.1 Experimental scheme

(1) Experimental site: Yan’an University’s College of Life Sciences has designated the field test station for agricultural ecosystems as the study’s location. The station is situated at 36°54′21′′N, 109°35′45′′E in the Baota district of Yan’an, Shaanxi Province. It is an average rain-fed dry agricultural area with 540mm of precipitation annually, mostly concentrated in July through September, with an average temperature of 8.7°C, 2421 hours of sunlight annually, and 146–179 days of frost-free time annually. The test region’s loess parent material is mostly yellow loamy soil, generally homogenous in kind. It is also extensively exposed on the ground. Sandy loam is a kind of soil (Chen et al., 2015). The pH of 8.6 in the soil layer ranging from 0 to 100 cm, 6.33 g kg^-1^ of organic matter, 0.88 g kg^-1^ of total nitrogen, 0.64 g kg^-1^ of total phosphorus, 19.72 g kg^-1^ of total potassium, 16.45 mg kg^-1^ of effective phosphorus, 145.28 mg kg^-1^ of fast-acting potassium, and uniform soil fertility are the basic physical and chemical properties of the soil.

(2) Single-factor randomized block design was used as the experimental design. Using the reference threshold of deficiency supplementation and the deficit and surplus criteria of maize demand for Ca and Mg, three Ca levels (none: 0.00kg hm^-2^, low: 17.50kg hm^-2^, high: 49.00kg hm^-2^) and three Mg levels (none: 0.00kg hm^-2^, low: 24.50kg hm^-2^, high: 35.00kg hm^-2^) were designed for the entire reproductive period of maize (Piao, 2020). For a total of 15 trial samples, five treatments with varying Ca and Mg levels were established and duplicated three times each. Low calcium (CA1), high calcium (CA2), low magnesium (MG1), high magnesium (MG2), and no calcium and no magnesium (CTL) were the specific treatments. To guarantee the correctness of the results, protection rows were placed on the edges of each test plot at a distance of 5 m, and the sample plots were spaced 4 m apart. The nutrients Ca and Mg were extracted from sugar alcohol chelated Ca (Ca≥180g L^-1^, 250g/bottle) and Mg (Mg≥120g L^-1^, 300g/bottle), which were non-toxic, easily absorbed, and beneficial to the environment. As the spring maize variety in testing, “H6281” was chosen because it is disease-resistant, high-yielding, highly adaptable, and quickly dehydrates seeds (Hu et al., 2016).

Ca and Mg supplementation schedule and methodology: As per previous studies (Rhodes et al., 2018), Ca and Mg were supplied during the subsequent phases of maize growth: silking-filling, elongation-tasseling, tasseling-silking, and seedling-elongation by the ratio of 1:2:3:4. From the seedling stage to the filling stage, this generated four nutrient gradients that were utilized to investigate continuously the dynamic characteristics of the physiological process by which Ca and Mg regulate the generation of STC in reproductive-stage maize kernels. Ca and Mg were chosen for uniform foliar spray distribution on the growth sites of above-ground organs, including leaves (including center leaves), stems, and kernels, after 16:00 hours on a windless and sunny day, to ensure that maize would adequately assimilate the nutrients.

(3) Field management: The experimental design comprised 15 sample sites, each measuring 6 m×7 m. Each sample plot was cultivated using full mulching technology, in which maize was applied directly onto the furrow surface. The mulch, composed of oxidized biodegradable ecological material, measured 0.008 mm in thickness. Every individual sample plot was established at a density of 60,000 plants hm^-2^. The furrow widths were 30 cm, monopoly heights were 15 cm (with a 10cm marginal monopoly width), and monopoly heights were 20 cm. The distance between the maize on the side of each row and the edge of the sample plot is 10 cm. Between rows, the maize was spaced at 50 cm. The seedlings were harvested on September 25, 2022, after being sowed on April 28, 2022. Prior to sowing, basal fertilizer was uniformly distributed in all sample sites in the following proportions: N, 130 kg hm^-2^; P_2_O_5_, 120 kg hm^-2^; K_2_O, 38 kg hm^-2^). This practice adhered to local recommendations that prioritized water conservation, yield stability, and fertilizer efficiency (Zheng et al., 2018). Sugar alcohol Ca and Mg fertilizers were applied topically on May 30th, June 25th, July 10th, and July 29th. Preserved maize kernels were collected and analyzed for indicator purposes on the following days after maize pollination: 8, 16, 24, 32, 40, and 48. Taking into account the impact of precipitation days, the precise dates of sample collection were as follows: August 7th, August 15th, August 21st, September 2nd, September 11th, and September 24th. Four replicates of each treatment were selected at random for each sampling.

### 2.2 Measurement method of main indicators

Considering the impact of variations in precipitation, 10g of representative leaves and kernels, separately, were collected at random from four maize plots (specifically, non-marginal plants were chosen to ensure the reliability of the test data) on September 7th, August 15th, August 21st, September 2nd, September 11th, and September 24th. Each sample plot contained healthy, disease-free growth. Individually labeled and sealed in tinfoil, clean gauze, and aluminum foil tape, kernel samples for each treatment were combined. After being promptly frozen in a liquid nitrogen tank, the specimens were returned to the laboratory where they were stored as a backup sample for measurement in an ultra-low temperature refrigerator set to -80°C. Biochemical experiments conducted indoors yielded the subsequent principal physiological and biochemical indicators: (1) endogenous hormones of maize leaf; (2) key enzymes of STC synthesis in maize kernel. Meanwhile, a random sampling technique was employed to collect 5 g of fresh, disease-free leaves from four non-marginal maize plots. Prior to being dried at 100°C to a constant weight, the grains were desiccated naturally after being thoroughly mixed. Save maize leaves that have been ground and crushed with an automatic ball mill and a 60-mesh sieve for Ca and Mg analysis.

Furthermore, 10 g of fresh, normal kernels were collected from four maize plants at the same time. These kernels were mixed and sealed in a preserving apparatus (a refrigerated icebox) before being returned to the laboratory in a timely manner. The samples underwent a natural drying process. Once the moisture content of the samples was reduced to 14% ± 1%, the kernels were dried in an oven set at 60°C until they reached a constant weight. The kernels were then weighed and tested for analysis using the Richards equation to determine the parameters of kernel filling in accordance with the method described by Khan et al. (2019). The kernels underwent a process of crushing, sieving via a 100-micron sieve, and subsequent storage in a desiccator in order to ascertain their respective contents of amylose, amylopectin, and total STC. Each of the aforementioned measures was examined four times for each indicator.

(1) Leaf Ca and Mg levels, using the method of HNO_3_-HClO_4_ ablation.

A single determination was conducted using 1g of maize leaf sample powder (with an accuracy of 0.0001g). Following a series of procedures including hydrochloric acid digestion, chilling, filtration, and HNO_3_-HClO_4_ elimination, the sample powder was transferred to a 100mL volumetric vial and thoroughly mixed. Following the pipetting of 10 mL of the sample solution and the addition of 0.50 mL of the internal standard solution (100 μg L^-1^) via pipette, the Ca and Mg levels in the leaves were determined using the filtrate in conjunction with an inductively coupled plasma emission spectrometer (ICP, AES-iCAP6300, Thermo Fisher Scientific, MA, USA) (Khan et al., 2010).

(2) Leaf ABA and IAA level, using the method of high-performance liquid chromatography (HPLC).

A 5g (0.001g) sample of crushed maize leaf was precisely weighed in a single determination, transferred to a 100mL volumetric flask, to which 80mL of methanol was added, and subsequently extracted using ultrasonic shaking for 20 minutes, vortexing for 2 minutes, and fixing with methanol as the extract solution. Subsequently, the ethyl acetate layer was dissolved in 10mL water, rotary evaporated to dry at 40°C, and extracted with 50mL ethyl acetate. The pH was adjusted to approximately 2 using a 1mol L^-1^ hydrochloric acid solution. Subsequently, the substance was dissolved in methanol, the volume was adjusted to 1 mL, it was filtered through a 0.45 μm membrane filter, and an analysis was conducted using liquid chromatography (HPLC) (Ahmad et al., 2019).

(3) The determination of maize kernel STC content was referred to the two-wavelength method of Khan et al. (2019).

The absorbance values of the test group samples were determined at 460 nm, 550 nm, 630 nm, and 740 nm, respectively, using the sample blank as a control. △A (amylose) = A630-A460, △A (amylopectin) = A550-A740, and the contents of amylose and amylopectin in the samples were calculated by the standard curves of amylose and amylopectin. Total starch content = amylose content + amylopectin content.

(4) For the determination of key enzyme activities of STC synthesis, 5 ml of Hepes-NaOH buffer (pH 7.5) was added to the kernel samples and ground in an ice bath. 30 µl of homogenate was taken, 1.8 ml of buffer was added, microcentrifuged, and the precipitate was suspended in buffer and used for the determination of GBSS activity. The rest of the homogenate was centrifuged at 10,000 g for 15 min, and the supernatant was used for the determination of SS, ADPG, SBE, and DBE activities (Dai, 2010). UDPG and SSS were determined according to the method of (Teng et al., 2015). All parameters were measured four times.

### 2.3 Analysis software and methods

SPSS 19.0, Origin Pro 2021, R 3.5.2, and Matlab 7.0 were utilized as scientific software for the statistical analysis of the experimental data. After maize pollination, the differences between all treatments in leaf Ca, Mg, endogenous hormones, enzymes of STC synthesis, STC, and its components were compared using one-way analysis of variance (ANOVA) followed by Tukey’s test. Using Pearson’s correlation analysis, the degree of correlation between Ca, Mg, and endogenous hormones for each treatment was determined at a significance level of P0.05. The redundancy analysis was employed to ascertain the extent to which Ca, Mg, endogenous hormones, and enzymes involved in STC synthesis impacted STC and its components. By employing the structural equation model, the primary driving pathway of STC accumulation and synthesis was identified. Using the least squares method and partial least squares method, linear regression and dominant regression models between STC and key driving indicators were constructed.

## 3 Results and analysis

### 3.1 Levels of Ca and Mg, endogenous hormones and their relationship in maize leaves

**Figure 1:**
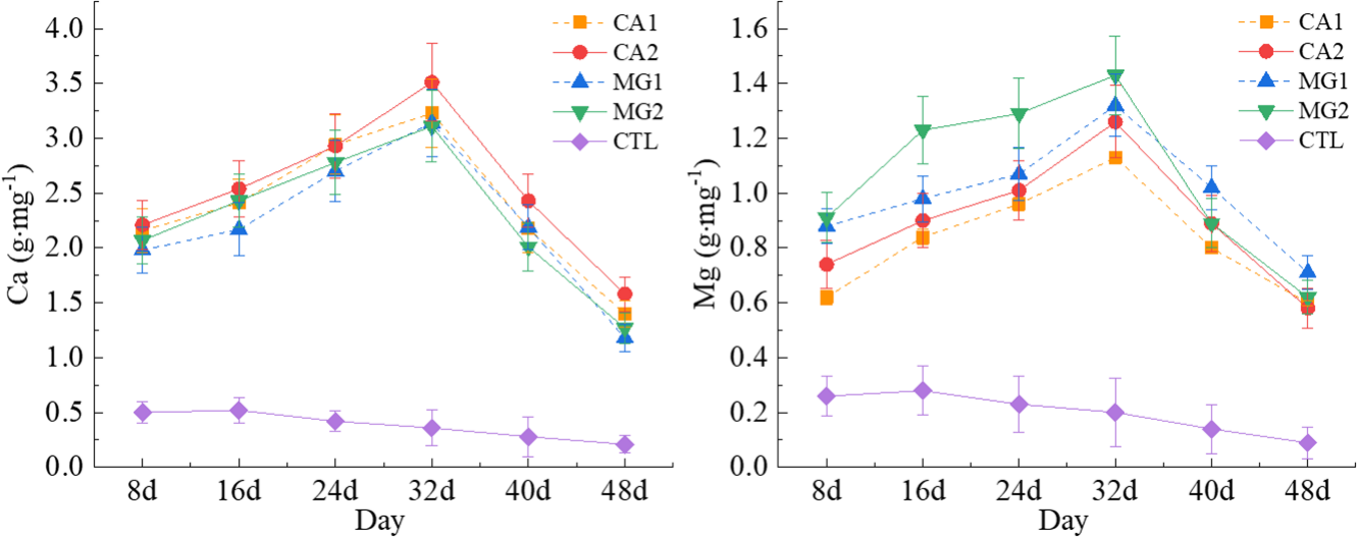
Characteristics of changes in leaf Ca and Mg levels after pollination of maize. Data represent mean ± standard deviation (n=4). CA1, CA2, MG1, MG2, and CTL represent the treatment of low Ca, high Ca, low Mg, high Mg, and no Ca and no Mg (the same below).

The levels of Ca and Mg in maize leaves exhibited a cyclical pattern of elevation and subsequent decline after the application of Ca and Mg, peaked on the 32nd day following pollination, and subsequently declined substantially as the reproductive period progressed. The levels of both Ca and Mg in CTL exhibited a persistent downward trajectory. The leaf Ca levels were most pronounced in CA2 and differed significantly from the other treatments (P<0.05) (**Figure 1**). MG1 and MG2 exhibited comparable Ca levels with no statistically significant distinctions; in contrast, CTL demonstrated the lowest Ca levels and differed significantly from the remaining four treatments (P<0.01). Between days 8 and 48 post-pollination, leaf Mg levels were found to be maximum in MG2 and significantly different from the other treatments (P<0.05); they were lowest in CA1. CTL exhibited the lowest level of Mg, which was notably reduced in comparison to the other treatments (P<0.01) (**Figure 1**).

**Figure 2:**
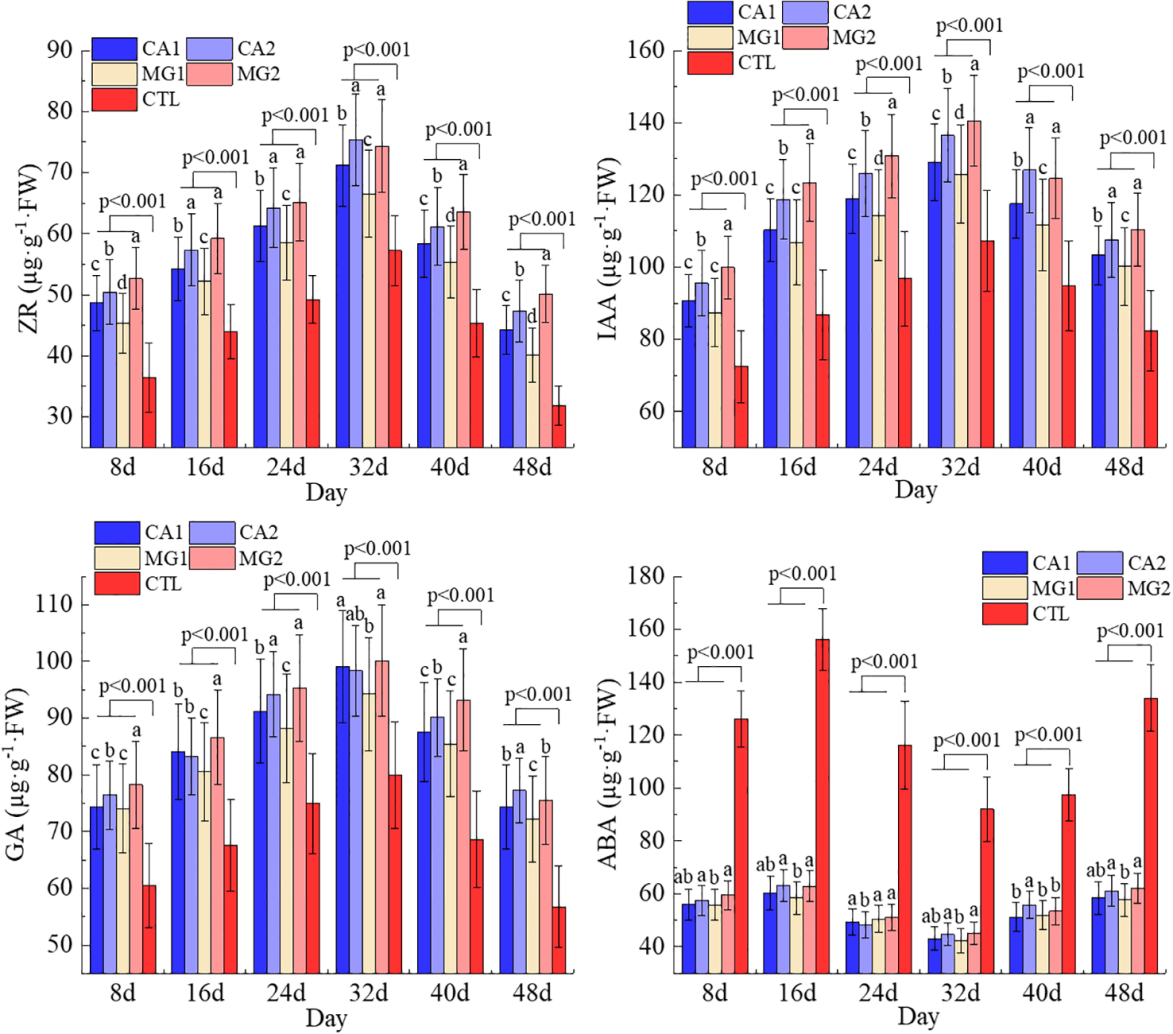
Characterization of endogenous hormone changes in leaves of maize after pollination. Data represent mean ± standard deviation (n=4). Lowercase letters a, b, c, d, and e represent the significance of the difference on the same day after maize pollination for each indicator at the P<0.05 level according to ANOVA followed by Tukey’s test. P<0.001 indicates that the treatment with added calcium and magnesium differed significantly from the control group. ABA, IAA, GA, and ZR represent abscisic acid, indole-3-acetic acid, gibberellin, and zeatin riboside, respectively.

Since the pollination, the IAA, GA, and ZR concentrations in maize leaves have exhibited a general pattern of initial increase followed by subsequent decrease. The highest value was attained on the 32nd day following pollination, while the lowest value was attained on the 48th day. The leaf ABA content exhibited a declining and then ascending trend, with its minimum value occurring 32 days following pollination. In contrast, the trend of ABA was observed to be precisely opposite that of IAA, GA, and ZR (**Figure 2**).

Following the results obtained regarding the effects of Ca and Mg supplementation levels on endogenous hormones, Ca and Mg significantly increased leaf IAA, GA, and ZR levels while decreasing leaf ABA levels. An elevation in the levels of IAA, GA, and ZR would substantially stimulate the division and elongation of cells in the root stem and leaf, augment the rate of pollination, and facilitate the growth and development of the kernel, in addition to the synthesis of nutritional indices. Conversely, a reduction in the level of ABA would attenuate the inhibitory impact on maize growth and postpone the start of maize senescence (**Figure 2**).

In terms of endogenous hormone variability among treatments, MG2 had the highest levels of endogenous hormones ZR, IAA, and GA in its leaves, followed by CA2. In contrast, MG1 and CA1 had the lowest levels of these hormones in their leaves, and the differences between them were statistically significant (P<0.05). The variations in ABA levels between the treatments supplemented with Ca and Mg were insignificant (**Figure 2**). The levels of endogenous hormones in CA1, CA2, MG1, and MG2 were all significantly different (P<0.001) with CTL.

**Figure 3:**
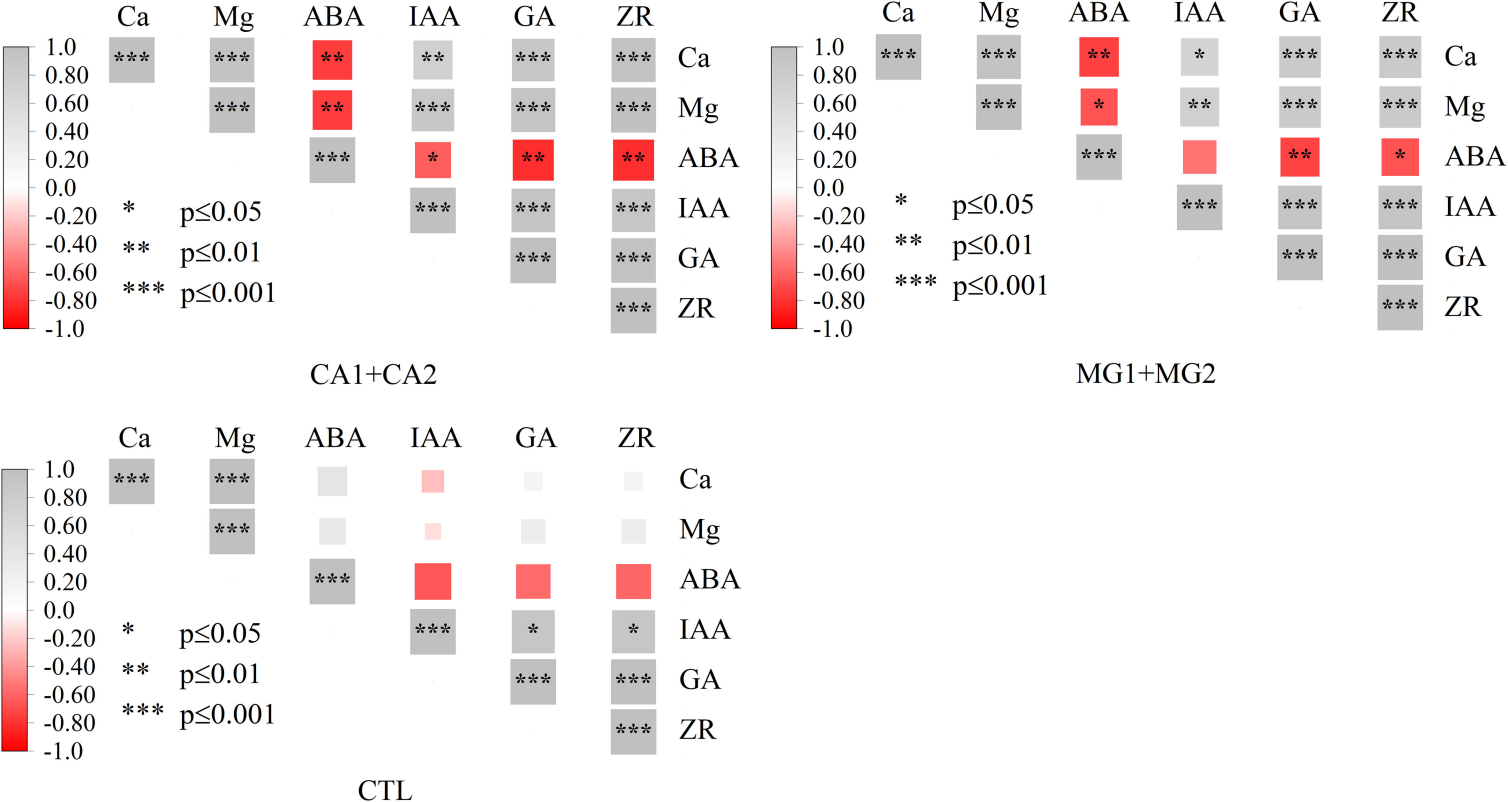
Correlation of Ca and Mg and endogenous hormones in maize leaves. *, **, *** represent P ≤ 0.05, P ≤ 0.01, P ≤ 0.001, respectively.

Following Ca and Mg supplementation, the levels of these two elements in leaves exhibited a highly significant positive correlation, as determined by the correlation analysis. Concurrently, noteworthy associations were observed between Ca and Mg and endogenous hormones; specifically, they exhibited substantial positive correlations with IAA, GA, and ZR and highly significant negative correlations with ABA. No statistically significant correlation was observed between Ca and Mg and endogenous hormones in CTL (**Figure 3**).

### 3.2 Characterization of changes in STC and enzymes of STC synthesis in maize kernels

**Figure 4:**
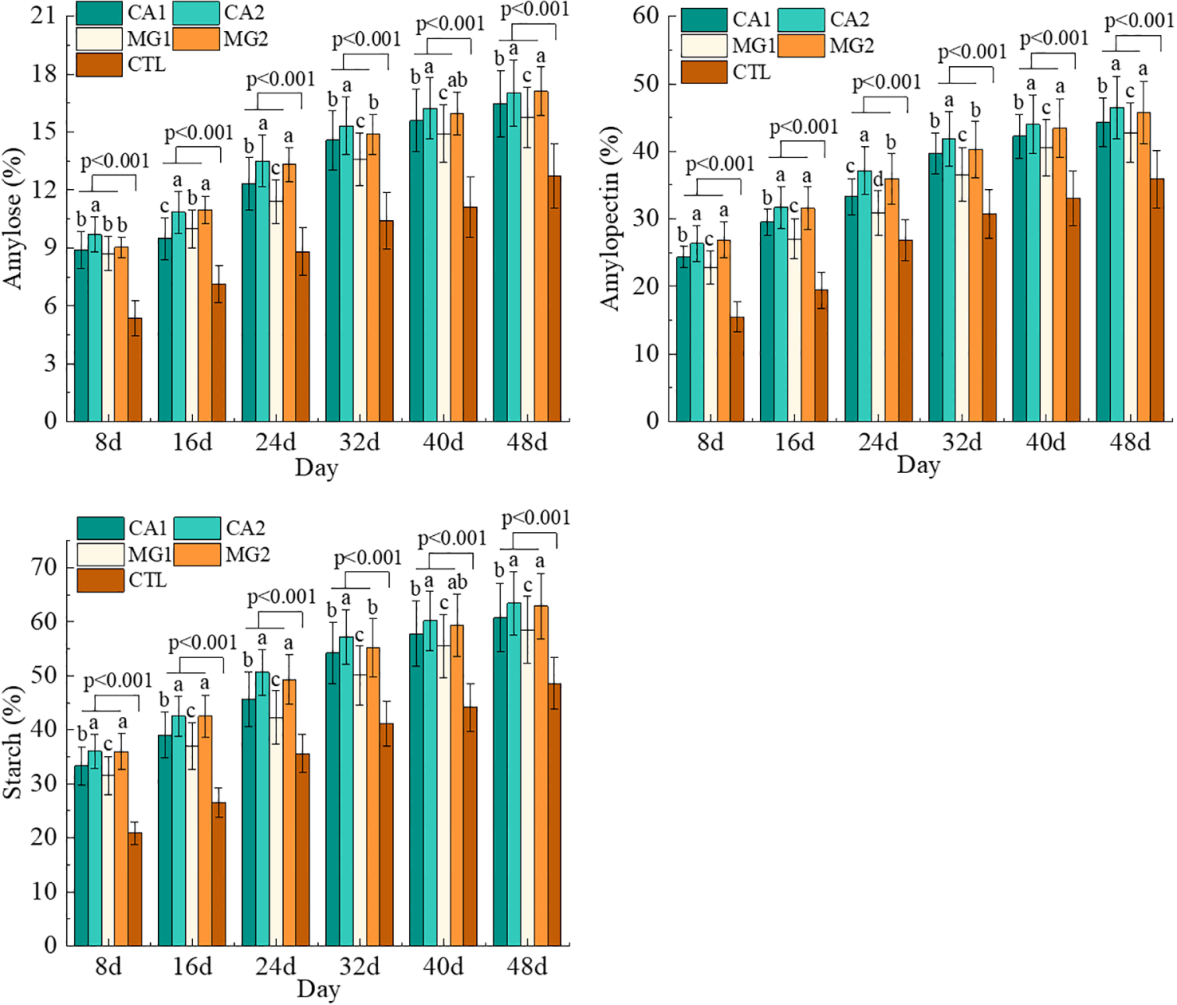
Characteristics of changes in the content of STC and its components in maize kernels after pollination. Data represent mean ± standard deviation (n=4). Lowercase letters a, b, c, d, and e represent the significance of the difference on the same day after maize pollination for each indicator at the P<0.05 level according to ANOVA followed by Tukey’s test. P<0.001 indicates that the treatment with added calcium and magnesium differed significantly from the control group.

Generally, the kernel STC and its component content continued to increase with the advancement of the reproductive stage after Ca and Mg supplementation. Notably, these values were significantly (P<0.001) greater than those of the CTL (**Figure 4**). Concerning the variability of STC throughout treatments, CA2 and MG2 exhibited the highest levels of kernel STC, amylose, and amylopectin, followed by CA1 and MG1. This finding suggests that the addition of Ca and Mg supplements resulted in a significant and moderate increase in the level of kernel STC. Furthermore, as the level of Ca and Mg added increased, so did the content of STC (**Figure 4**).

**Figure 5:**
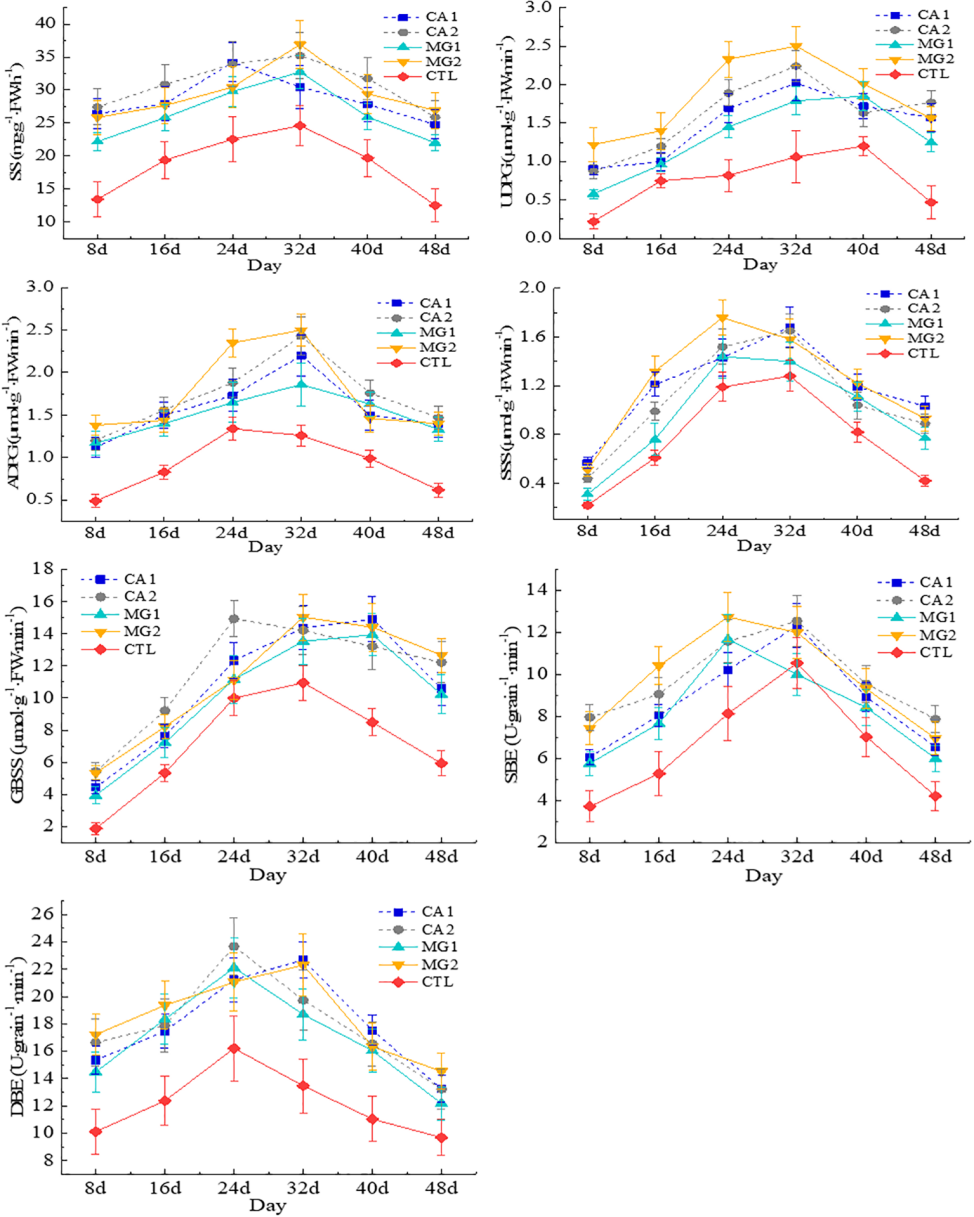
Characterization of changes in kernel enzymes of STC synthesis after pollination in maize. Data represent mean ± standard deviation (n=4). SS, UDPG, ADPG, SSS, GBSS, SBE, DBE represent sucrose synthase, uridine diphosphate-glucose pyrophosphorylase, adenosine diphosphate-glucose pyrophosphorylase, soluble starch synthase, granule-bound starch synthase, starch branching enzyme, and starch debranching enzyme, respectively (the same below).

The enzyme activities involved in STC synthesis, encompassing SS, UDPG, ADPG, SSS, GBSS, SBE, and DBE, exhibited a pattern of initial increase followed by subsequent decrease in response to Ca and Mg supplementation. The enzyme activities peaked on the 24th and 32nd day following maize pollination (**Figure 5**). Treatments CA1, CA2, MG1, and MG2 exhibited significantly higher enzyme activities for STC synthesis than CTL. This suggests that the addition of Ca and Mg significantly increased the activity of enzymes involved in STC synthesis. Based on the observed variation in enzyme activities during STC synthesis in all treatments, it was determined that MG2 contained the highest activities of UDPG, ADPG, SSS, and SBE; CA2 had the highest activities of SS and GBSS; and MG1 had the lowest activities of all the aforementioned enzymes (**Figure 5**). In summary, the enzyme activities involved in STC synthesis exhibited a greater magnitude in treatments supplemented with Ca and Mg, with MG2 and CA2 exhibiting the highest value, followed by CA1, and MG1.

### 3.3 Physiological mechanisms of Ca and Mg regulation of STC synthesis

**Figure 6:**
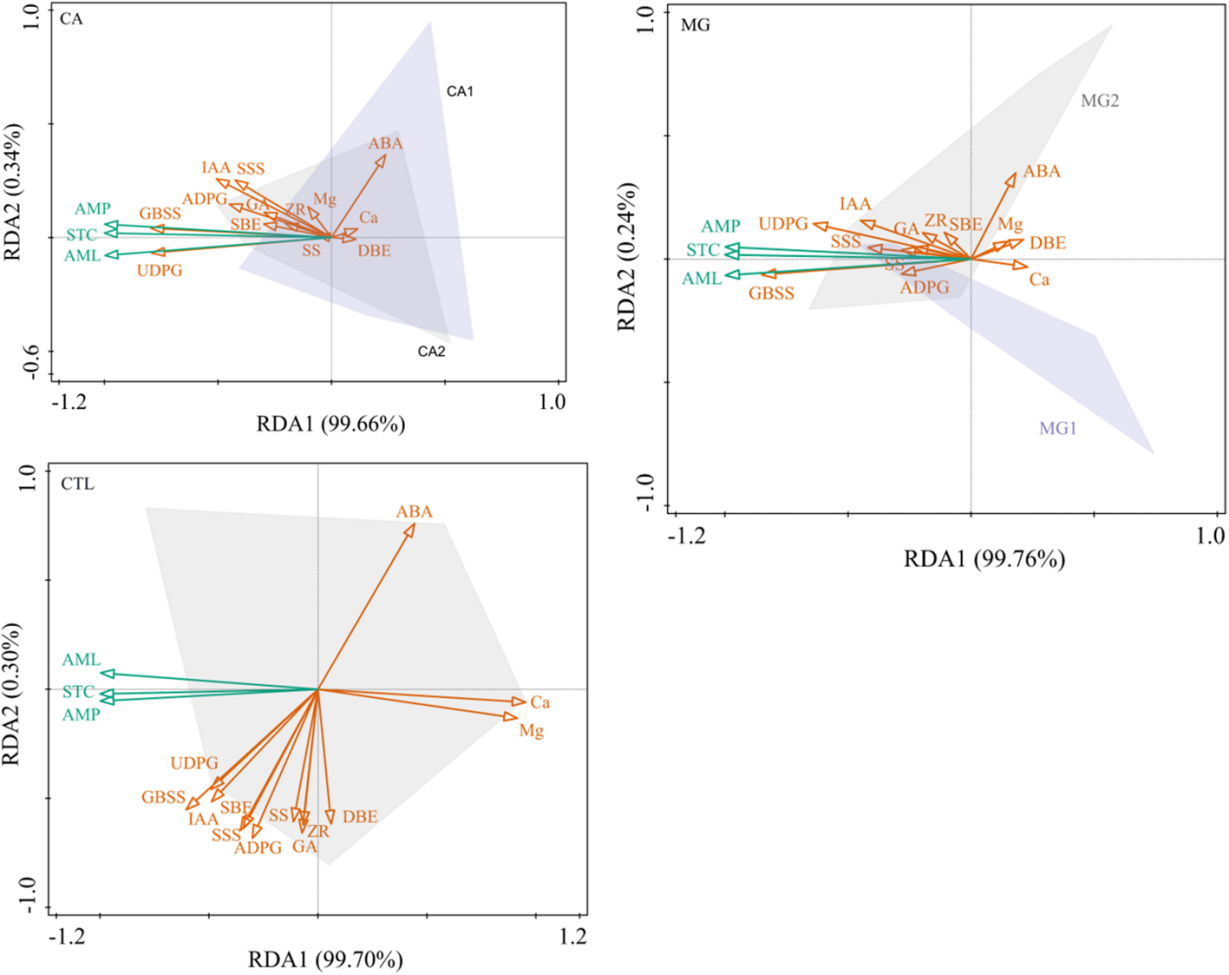
Redundancy analysis of physiological indicators regulating the accumulation of STC synthesis in maize kernels. STC, AML, and AMP represent starch, amylose, and amylopectin, respectively (the same below).

After conducting a redundancy analysis, it was determined that the primary endogenous hormone factors influencing the synthesis of STC and its components in the kernel subsequent to Ca and Mg supplementation were IAA and ABA. The principal synthase enzymes controlling the synthesis of STC were UDPG, GBSS, and SSS. It is worth noting that the impact of the key enzymes of STC synthesis was more pronounced than that of the endogenous hormones on the STC and its components (**Figure 6**). Furthermore, it was observed that STC, AML, AMP, and ABA exhibited negative correlations with these primary drivers, whereas positive correlations were found with all other significant factors. STC synthesis in CTL was significantly more influenced by leaf Ca and Mg levels than by endogenous hormones and key enzymes of STC synthesis; however, STC, AML, and AMP exhibited negative correlations with STC (**Figure 6**).

Overall, Ca and Mg supplementation attenuated the extent to which leaf Ca and Mg levels affected kernel STC, while contributing to the more pronounced driving effect of IAA and ABA on STC, and also to the significant positive regulation of STC synthesis by UDPG and GBSS.

**Figure 7:**
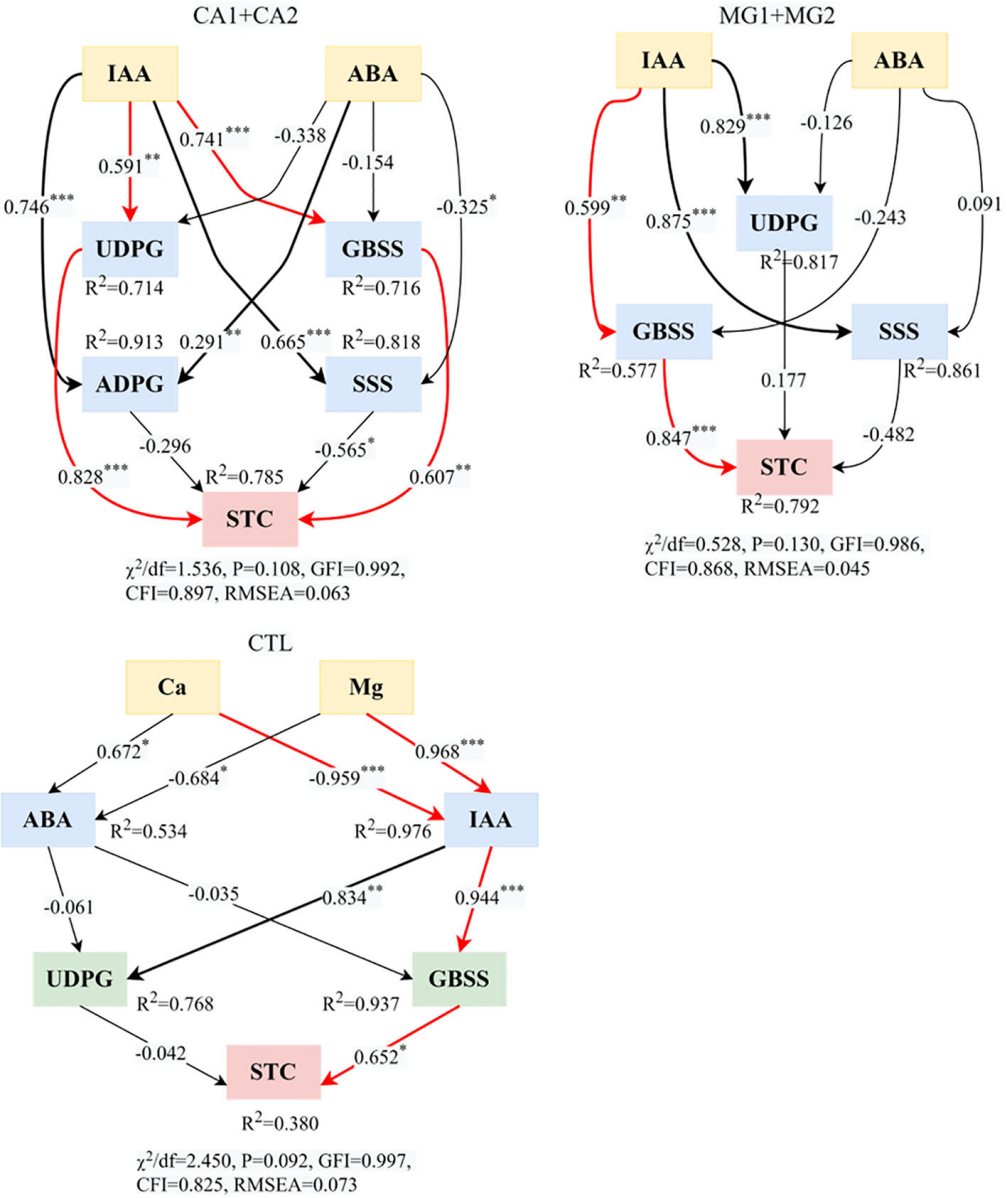
Structural equation modeling of Ca and Mg-regulated physiological processes of STC synthesis in maize kernels. ^*^ represents P<0.05, ^**^ represents P<0.01, ^***^ represents P<0.001. χ^2^ represents the chi-square; df represents the degree of freedom; GFI represents the goodness of fit index; CFI represents the comparative fit index; RMSEA represents the root mean square error of approximation.

Based on the structural model of physiological regulation of kernel STC, “IAA–UDPG or GBSS–STC” (**Figure 7**) was the predominant physiological regulatory pathway by which Ca supplementation affected STC synthesis. Regarding path coefficients, it was observed that IAA had a highly significant positive impact on both UDPG and GBSS. Furthermore, both UDPG and GBSS exhibited highly significant positive influence relationships on STC. “IAA–GBSS–STC” was the predominant physiological regulation pathway of STC synthesis in reaction to Mg supplementation. Positive effects were observed for both the regulation of STC by GBSS and the impact of IAA on GBSS; this pathway was identical to that of the treatment CTL (**Figure 7**). According to the aforementioned findings, IAA and GBSS were two fundamental physiological indicators that controlled the accumulation of STC synthesis in kernels.

**Figure 8:**
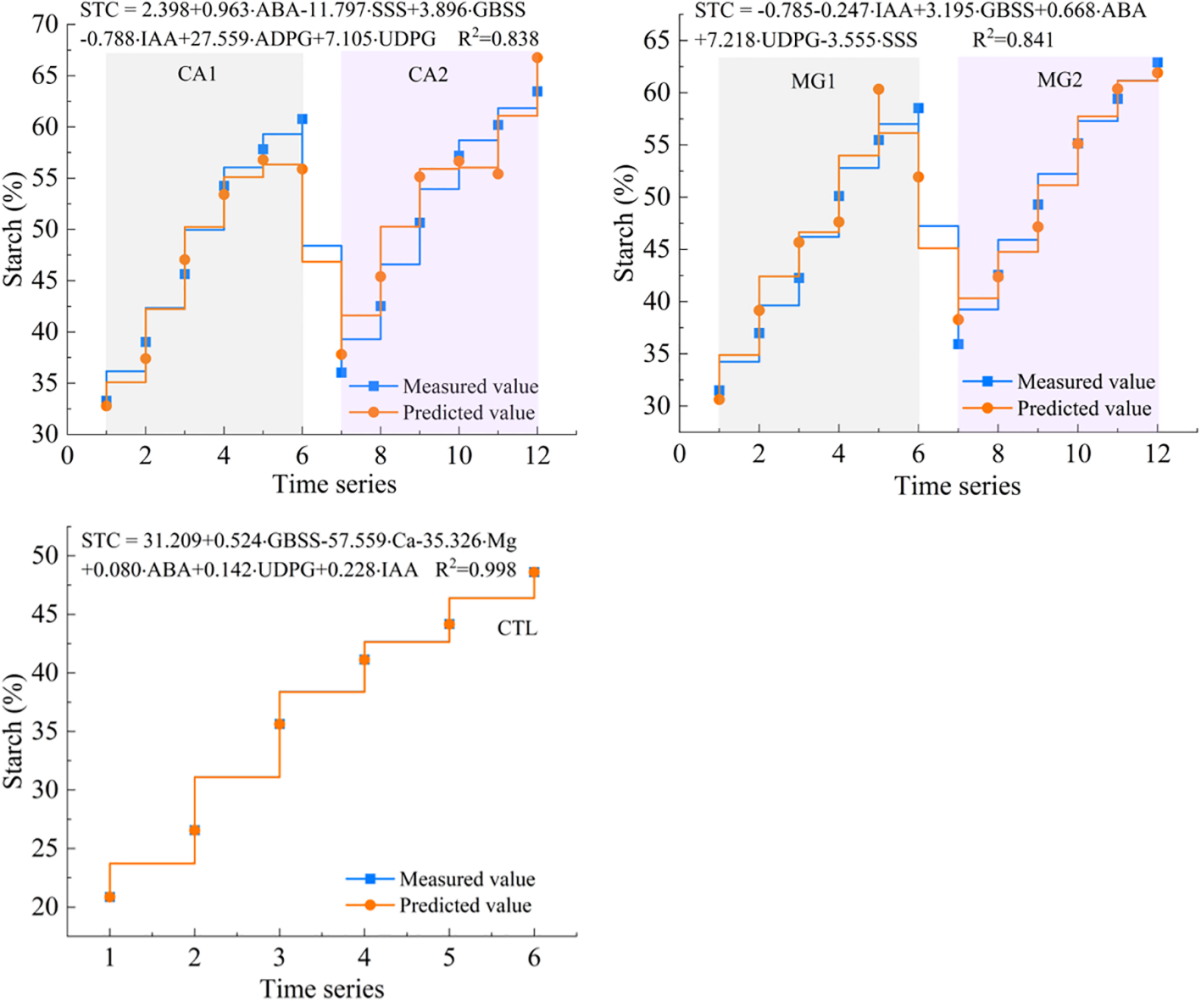
Curve fitting of dominant drivers to kernel STC levels.

By integrating the results of redundancy analysis and employing partial least squares estimation to simulate the accumulation of kernel STC, the dominant driving indicators of STC synthesis were utilized to assess the level of accumulation. The multiple regression function between these variables was assessed, and it was observed that all fitted equations had R^2^ values exceeding 0.80. This finding suggests that the aforementioned core driving indicators adequately characterized the accumulation and synthesis of kernel STC (**Figure 8**).

## 4 Discussion

### 4.1 Effects of Ca and Mg supplementation on Ca and Mg levels and endogenous hormones in maize leaves

Ca and Mg are crucial nutrients for the development and growth of crops. The supplementation of Ca and Mg into maize leaves resulted in an initial increase in Ca and Mg levels, followed by a decrease as pollination progressed fertility-wise (**Figure 1**). This may be because maize requires more nutrients to support the development of flower kernels and ovules following pollination, therefore, Ca and Mg levels will increase initially. The maturation of the flower kernels and ovules during the reproductive period leads to a reduction in the maize’s demand for Ca and Mg, consequently causing levels to decline (Cakmak and White, 2020). Both Mg and Ca exhibit highly significant positive correlations in maize, indicating that they have an interactive relationship. The increase in Ca levels leads to a corresponding rise in Mg levels in maize leaves, primarily as a result of the reciprocal influence between Ca and Mg (Ciampitti and Vyn, 2013). Ca is a structural and functional element that maintains the stability of maize cells and participates in cell wall synthesis. Elevated Ca concentrations stimulate the development of cell walls and bolster cellular mechanical integrity, leading to the production of maize leaves that are more robust and stable (Tang and Luan, 2017). Additionally, Ca regulates the transport and absorption of Mg^2+^ in maize. The formation of complexes between Ca^2+^ and specific proteins within the cell can facilitate the absorption and transport of Mg^2+^. By controlling the activity of these complexes, the efficiency of their uptake and utilization by the maize can be enhanced (Collignon et al., 2011). Ca levels increase concurrently with Mg. This is because Ca is typically present in maize in ionic form and is transported across the cell membrane via Ca channels (Rhodes et al., 2018). By influencing the activity of these Ca channels, Mg can increase both the rate and quantity of Ca translocation. Additionally, Ca is deposited as Ca in the interstitial spaces and cell walls to form a Ca matrix. Ca deposition can be facilitated by the influence of Mg on the formation and stability of the Ca matrix (Ertiftik and Zengin, 2017). Additionally, numerous enzymes in maize utilize Mg as a cofactor, which regulates the activity and function of these enzymes. Among these enzymes are those that facilitate the transport, utilization, and absorption of Ca. Consequently, elevated Mg concentrations may facilitate the uptake and utilization of Ca, resulting in a concomitant rise in Ca levels (Farhat et al., 2016a).

The synthesis of nutritional quality in maize is significantly impacted by endogenous hormones, which can also regulate physiological processes, growth and development, nutrient uptake and transport, and maize metabolism (Malaga et al., 2020). Ca and Mg supplementation substantially increased the leaf concentrations of IAA, GA, and ZR while decreasing the leaf concentrations of ABA. In leaves, Ca and Mg exhibited a positive correlation with IAA, GA, and ZR, while they demonstrated a negative correlation with ABA (**Figures 2**, **3**). This can be attributed to their distinct impacts on the growth and development of maize. Ca and Mg are integral components in maize internal signaling and are crucial nutrients for maize development and growth. According to Modareszadeh et al. (2020), the administration of Ca and Mg supplements improves the maize’s receptivity to phytohormones, including IAA, GA, and ZR, which stimulate the growth and development of maize. In maize, these hormones govern cellular processes including elongation, differentiation, and cell division. The growth of maize is stimulated by the activity of these hormones, which is enhanced by the presence of Ca and Mg (Naeem et al., 2020). Conversely, ABA is a hormone that primarily regulates the maize plant’s response to stress and adversity and inhibits maize growth. The inhibitory impact of ABA on maize growth is mitigated through the reduction in synthesis and accumulation of ABA due to the availability of Ca and Mg (Piao, 2020). This implies that Ca and Mg exert a beneficial influence on the control of maize development and growth.

### 4.2 Effect of Ca and Mg supplementation on STC synthesis in maize kernels and its driving mechanism

The percentage of STC and components in maize kernels continued to rise following Ca and Mg supplementation. This is because supplementation with Ca and Mg can influence the process of carbon metabolism in maize. More precisely, the involvement of Ca and Mg in the synthesis and transportation of sucrose in maize has been observed to impact the synthesis and accumulation of STC (Farhat et al., 2016b). Ca and Mg regulate the synthesis and translocation of sucrose, which is a precursor to STC. Sucrose synthesis and translocation are enhanced in maize kernels when Ca and Mg are abundant, thus facilitating STC accumulation and synthesis (Ferreira et al., 1999). Supplementation with Ca and Mg increased the activity of STC synthesis enzymes (**Figure 5**). The reason for this is that Ca and Mg function as coenzymes, facilitating enzyme activity within maize cells. Both substances can form enzyme-metal ion complexes with STC synthesis enzymes, consequently augmenting the enzyme’s catalytic activity (Kaya et al., 2018). This phenomenon occurs due to the interaction between the positively charged residues in the enzyme and the negatively charged Ca^2+^ and Mg^2+^. This interaction facilitates the formation of the active site and enhances the stability of the enzyme’s steric structure (Piao, 2020). Furthermore, the catalytic efficiency of the enzyme can be enhanced by regulating the substrate binding and release processes with the assistance of Ca and Mg (Rosanoff and Kumssa, 2020). Therefore, Ca and Mg supplementation can promote the synthesis and accumulation of STC by increasing the activity of enzymes involved in STC synthesis in maize kernel.

The primary endogenous hormonal factors that influence STC synthesis are IAA and ABA (**Figure 6**). By influencing the gene expression and activity of STC synthesis enzymes, IAA primarily participates in the regulation of STC synthesis (Yang et al., 2019). IAA can stimulate the post-transcriptional and transcriptional regulation of genes encoding enzymes responsible for STC synthesis, thereby increasing STC synthase expression. Furthermore, IAA can enhance the catalytic efficiency of STC synthesis by directly influencing the activity of enzymes (Zakari et al., 2020). Consequently, IAA significantly and positively regulated the synthesis of kernel STC (**Figure 7**). Conversely, ABA functions as an inhibitory hormone. The primary mechanism by which ABA regulates STC synthesis in maize is through the inhibition of transcriptional and post-transcriptional regulation of the gene-encoding enzymes involved in STC synthesis. By inhibiting and decreasing the expression of the genes encoding the enzymes involved in STC synthesis, ABA is capable of reducing STC synthesis. As a result, the catalytic efficiency of STC is diminished as ABA inhibits the activity of enzymes involved in its synthesis (Singh et al., 2020). The principal synthetic enzymes that control STC synthesis are UDPG, GBSS, and SSS (**Figure 6**). UDPG serves as the precursor material for STC synthesis and is the primary substrate for the process. Following the conversion of UDPG to glucose-1-phosphate, a sequence of enzyme-catalyzed reactions completes the synthesis of the STC molecule (Rahim et al., 2020). It catalyzes GBSS, one of the essential enzymes for STC synthesis. STC synthesis is catalyzed by GBSS, an essential enzyme that facilitates the polymerization of glucose molecules into STC chains. The primary structure of the STC molecule is formed of α-1,4-glucose chains, which are produced by GBSS from G1P (Han et al., 2022). An additional crucial enzyme in the synthesis of STC is SSS, which facilitates the polymerization of G1P into branched α-1,6-glucose chains. The degree of branching of the STC molecule is determined by the activity of SSS, this, in turn, influences the structure and properties of the STC (Guo et al., 2021). To summarize, the primary synthases that govern STC synthesis are UDPG, GBSS, and SSS. These enzymes participate in distinct stages of STC synthesis and collectively control their rate, structure, and properties (Jiang et al., 2013).

“IAA–UDPG or GBSS–STC” was the predominant physiological regulatory pathway for STC synthesis by Ca. IAA had a highly significant positive effect on both UDPG and GBSS, and GBSS and UDPG, in turn, had a highly significant positive effect on STC (**Figure 7**). This could potentially be attributed to the regulatory function of Ca^2+^ in plant cells, which includes signaling for hormones (Rashid et al., 2020). An increase in Ca^2+^ concentration induces interactions with intracellular proteins, resulting in alterations to the conformation and activity of said proteins. Ca^2+^ can enhance the beneficial regulatory effects of IAA on UDPG and GBSS when it interacts with the IAA signaling pathway (Lando et al., 2020). One possible mechanism by which Ca^2+^ enhances the ability of IAA to regulate the expression of target genes is by interacting with proteins in the IAA signaling pathway and facilitating the transmission of the signal transduction chain (Wei et al., 2015). In addition, an increase in Ca^2+^ can influence the subcellular localization and intracellular activity of enzymes. By controlling the translation, post-translational modification, or subcellular localization of UDPG and GBSS, it may augment the beneficial regulatory impact of IAA (Wang et al., 2014). The process of STC synthesis is intricate, requiring the involvement of numerous enzymes and regulatory factors. UDPG, which functions as a glucose-forming enzyme in the precursor of the amylopectin gene, is crucial for STC synthesis. GBSS plays a pivotal role in the biosynthesis of STC granules as an enzyme (Corbi et al., 2011). In the STC synthesis pathway, Ca^2+^ interacts with UDPG and GBSS to alter their conformation and activity, thereby promoting the STC synthesis process and enhancing the enzymes’ catalytic capability (Guo et al., 2023).

“IAA–GBSS–STC” was the predominant physiological regulatory pathway governing STC synthesis by Mg. Positive effects were observed, specifically in the regulation of STC by GBSS and the impact of IAA on GBSS (**Figure 7**). The beneficial impact of IAA on GBSS subsequent to Mg supplementation could potentially be attributed to the function of Mg as a crucial cofactor in the modulation of GBSS enzyme activity. A gigantic interaction between Mg^2+^ and the GBSS enzyme can regulate the enzyme’s stability and conformation (Roy et al., 2019). These structural alterations could potentially increase the GBSS enzyme’s vulnerability to interaction with IAA and amplify the impact of IAA on its activity. Furthermore, intracellular signaling pathways can have their ion concentrations and enzyme activities regulated by Mg^2+^, an essential participant in signaling (Peng et al., 2020). By, among other mechanisms, influencing the synthesis, degradation, or transport of the signaling molecule IAA, Mg^2+^ may augment the positive regulatory effect of IAA on GBSS. One potential explanation for the positive regulation of STC synthesis by GBSS via Mg^2+^ is that Mg^2+^ can interact with the GBSS enzyme to preserve the enzyme’s three-dimensional structure stability and proper folding state, thereby influencing the activity and conformation of the enzyme (Rehman et al., 2018). Mg^2+^ has the potential to regulate the catalytic efficiency and substrate binding affinity of GBSS enzymes through its binding to these enzymes. This, in turn, could enhance the activity of GBSS enzymes and facilitate the synthesis of STC. The level of GBSS gene expression may also be regulated by Mg^2+^, either directly or indirectly (Zhao et al., 2012). Elevated Mg^2+^ availability has the potential to stimulate or facilitate the transcription and translation of GBSS genes, consequently augmenting both GBSS expression and STC synthesis.

## 5 Conclusion

After Ca and Mg supplementation, the levels of these elements in maize leaves initially rose and subsequently fell. The peak levels of leaf IAA, GA, and ZR were observed on the 32nd day afterwards pollination, followed by a gradual decline. The lowest levels of leaf ABA were observed on day 32 following pollination. Supplemental Ca and Mg increased leaf IAA, GA, and ZR levels significantly while decreasing leaf ABA levels. Ca and Mg were correlated positively with leaf IAA, GA, and ZR, and negatively with ABA.

The supplementation of Ca and Mg resulted in a sustained increase in the level of STC and its components in maize kernels. Enzymes engaged in the synthesis of STC exhibited a substantial increase in activity, which was positively correlated with the gradient of Ca and Mg levels. The endogenous hormone factors IAA and ABA exhibited the greatest impact on STC synthesis, while the synthase enzymes UDPG, GBSS, and SSS demonstrated the most influence on STC formation. Regarding STC synthesis, “IAA–UDPG or GBSS–STC” is the principal regulatory pathway for Ca, whereas “IAA–GBSS–STC” is the principal regulatory pathway for Mg. Both UDPG and GBSS were positively influenced by IAA, and the regulation of STC by UDPG and GBSS was also positive.

## Data availability statement

The original contributions presented in the study are included in the article/supplementary material. Further inquiries can be directed to the corresponding author.

## Author contributions

ZH: Conceptualization, Data curation, Formal Analysis, Funding acquisition, Writing – original draft, Writing – review & editing. XS: Investigation, Methodology, Validation, Visualization, Writing – review & editing. TZ: Conceptualization, Project administration, Software, Supervision, Validation, Writing – review & editing. JY: Investigation, Methodology, Resources, Software, Supervision, Validation, Visualization, Writing – review & editing.
